# Targeting EHMT2 reverses EGFR-TKI resistance in NSCLC by epigenetically regulating the PTEN/AKT signaling pathway

**DOI:** 10.1038/s41419-017-0120-6

**Published:** 2018-01-26

**Authors:** Lihui Wang, Xiaoyu Dong, Yong Ren, Juanjuan Luo, Pei Liu, Dongsheng Su, Xiaojun Yang

**Affiliations:** 1 Department of Pharmacology, Shenyang Pharmaceutical University, 110016 Shenyang, China; 2 Department of Pathology, Wuhan General Hospital, People’s Liberation Army of China, Wuhan, China; 3 Center for Neuroscience, Medical College of Shantou University, 515041 Shantou, PR China

## Abstract

Epidermal growth factor receptor tyrosine kinase inhibitor (EGFR-TKI) resistance is a major obstacle in the treatment of non-small cell lung cancer (NSCLC). Epigenetic alterations have been shown to be involved in NSCLC oncogenesis; however, their function in EGFR-TKI resistance remains uncharacterized. Here, we found that an EHMT2 inhibitor, UNC0638, can significantly inhibit cell growth and induce apoptosis in EGFR-TKI-resistant NSCLC cells. Additionally, we also found that EHMT2 expression and enzymatic activity levels were elevated in EGFR-TKI-resistant NSCLC cells. Moreover, we determined that genetic or pharmacological inhibition of EHMT2 expression enhanced TKI sensitivity and suppressed migration and tumor sphere formation in EGFR-TKI-resistant NSCLC cells. Further investigation revealed that EHMT2 contributed to PTEN transcriptional repression and thus facilitated AKT pathway activation. The negative relationship between EHMT2 and PTEN was confirmed by our clinical study. Furthermore, we determined that combination treatment with the EHMT2 inhibitor and Erlotinib resulted in enhanced antitumor effects in a preclinical EGFR-TKI-resistance model. We also found that high EHMT2 expression along with low PTEN expression can predict poor overall survival in patients with NSCLC. In summary, our findings showed that EHMT2 facilitated EGFR-TKI resistance by regulating the PTEN/AKT pathway in NSCLC cells, suggesting that EHMT2 may be a target in the clinical treatment of EGFR-TKI-resistant NSCLC.

## Introduction

Non-small cell lung cancer (NSCLC) is the leading cause of cancer-related death worldwide^1^, and treatment failure in patients with the disease is usually attributable to the lack of effectiveness of traditional chemotherapeutic drugs, including platinum and paclitaxel, which mainly induce drug resistance in NSCLC cells^2^. A recent study showed that epidermal growth factor receptor tyrosine kinase inhibitors (EGFR-TKIs), such as Gefitinib or Erlotinib, may be effective anticancer therapeutic agents and that the indicated drugs may have beneficial clinical effects in patients with EGFR mutation-related cancer^3^. Most cancers with EGFR mutations initially display positive responses to EGFR-TKI treatment; however, the vast majority of these tumors ultimately become resistant to treatment and progress within a median time period of ~12 months^4^.

Two genetic mechanisms have been demonstrated to contribute to EGFR-TKI resistance in NSCLC. Secondary resistance-inducing mutations in the EGFR, which occur mainly at EGFR T790M, account for ~50% of cases of acquired EGFR-TKI resistance in NSCLC^5,6^. In addition, ~15–20% of cases of EGFR-TKI resistance have been shown to be associated with amplification of the *MET* or *HER2* gene, which subsequently activates intracellular signaling pathways downstream of the EGFR^6–8^. However, studies aiming to improve the understanding of the mechanisms contributing to EGFR-TKI resistance and to identify potential approaches to reversing EGFR-TKI resistance remain necessary.

Epigenetic phenomena, including DNA methylation and histone modification, have been reported to be involved in NSCLC development and progression^9–11^; however, the role of epigenetic modifications in EGFR-TKI resistance remains poorly understood. To investigate the epigenetic modifications underlying acquired EGFR-TKI resistance in NSCLCs, we administered a series of DNA methylation and histone modification enzyme inhibitors to Erlotinib-resistant NSCLC cells (NSCLC/ER). We found that only UNC0638, an inhibitor of the histone lysine methyltransferase EHMT2, significantly inhibited NSCLC/ER cell growth. Further study showed that EHMT2 expression and activity levels were upregulated in NSCLC/ER cells, suggesting that EHMT2 plays an important role in EGFR-TKI resistance in NSCLC. In addition, inhibiting EHMT2 expression not only reversed Erlotinib resistance in NSCLC/ER cells but also attenuated the malignant phenotype of NSCLC/ER cells. Moreover, our results demonstrated that EHMT2-mediated *PTEN* inhibition contributed to NSCLC/ER resistance. Notably, the combination of the indicated EHMT2 inhibitor and Erlotinib could robustly retard tumor growth in NSCLC/ER xenograft models by regulating the PTEN/AKT pathway. Furthermore, pathological analysis suggested that the balance between PTEN and EHMT2 expression may be a promising predictive biomarker for the prognoses of patients with NSCLC.

## Results

### A specific EHMT2 inhibitor significantly suppressed EGFR-TKI-resistant NSCLC cell growth

**Fig. 1:**
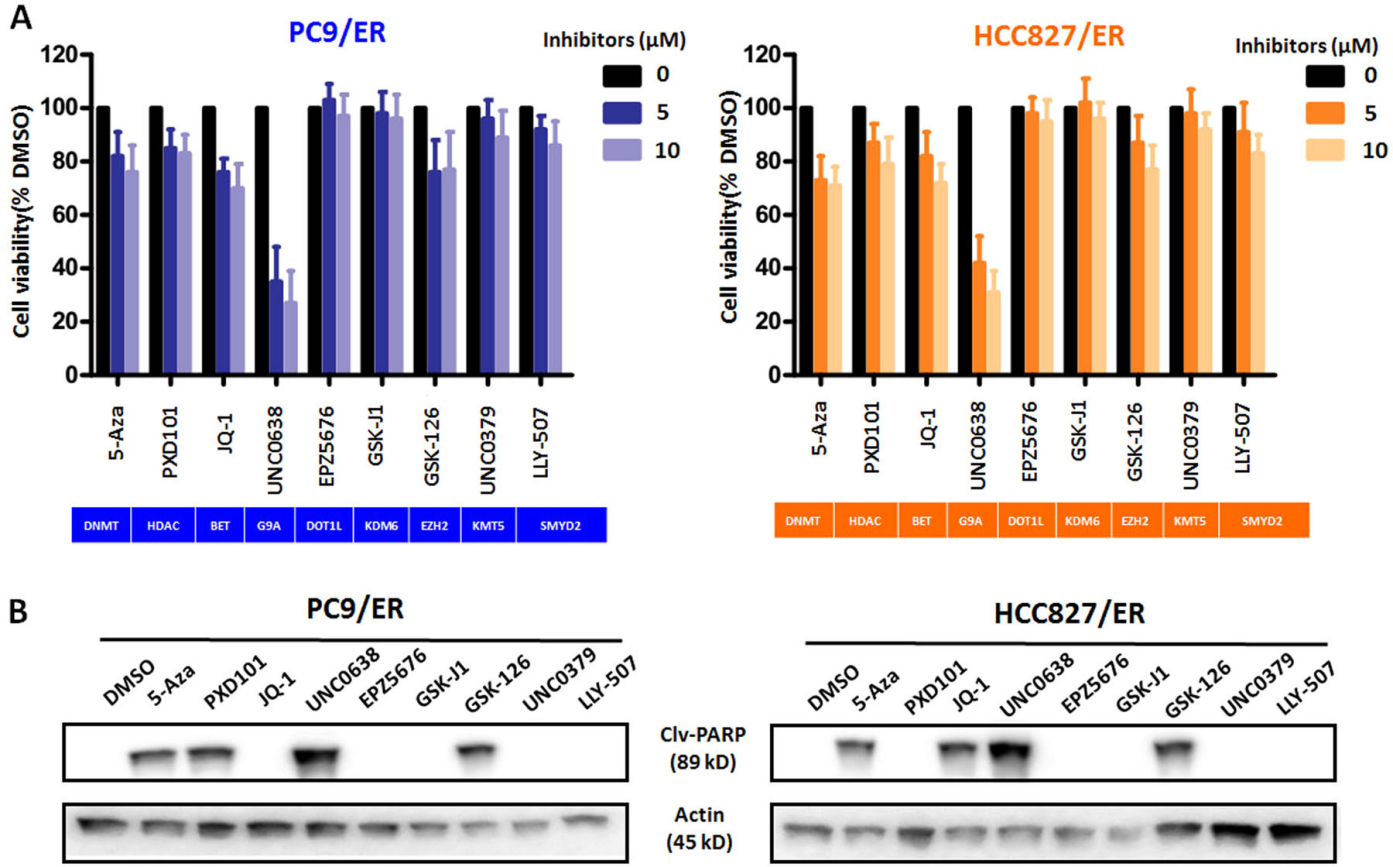
Effects of epigenetic enzyme inhibitors on cell growth and apoptosis in EGFR-TKI-resistant NSCLC cells **a** The growth of PC9/ER and HCC827/ER cells treated with epigenetic inhibitors at different concentrations (5 and 10 μM). Cell lines treated with DMSO were used as controls. **b** The effects of treatment with the indicated epigenetic inhibitors on cleaved PARP (Clv-PARP) expression in both PC9/ER and HCC827/ER cells. β-actin was used as a loading control

To elucidate the epigenetic mechanisms by which NSCLCs acquire resistance to EGFR-TKIs, we treated two NSCLC/ER cell lines, namely, the PC9/ER and HCC827/ER cell lines, with a series of epigenetic enzyme inhibitors at different pharmacological concentrations (0, 5, and 10 μM). As shown in Fig. 1a, treatment with 5-Aza (a DNMT inhibitor), PDX101 (a HDAC inhibitor), JQ-1 (a BRD4 inhibitor), and GSK126 (an EZH2 inhibitor) moderately inhibited cell growth in the indicated cell lines, whereas treatment with EPZ5676 (a DOT1L inhibitor), GSK-J1 (a KDM6 inhibitor), UNC0379 (a KMT5 inhibitor), and LLY507 (a SMYD2 inhibitor) had no significant effect on cell growth in the two cell lines. Notably, the EHMT2 inhibitor UNC0638 was extremely effective in inhibiting cell growth in both PC9/ER and HCC827/ER cells but showed a relatively weak inhibition in their parental cells (see Supplementary Fig. 1A), suggesting that EHMT2 plays an important role in EGFR-TKI resistance in NSCLC cells.

To elucidate the mechanisms responsible for the inhibitory effects of these agents on cell growth, we subsequently performed western blotting to detect the expression level of cleaved-PARP, an apoptosis biomarker, in the indicated inhibitor-treated resistant cell lines. Our data revealed that Cleaved-PARP expression was induced by treatment with 5-Aza, GSK-126, or UNC0638 (10 μM) but was not induced by treatment with EPZ5676, GSK-J1, UNC0379, or LLY507 (10 μM) (Fig. 1b and Supplementary Fig. 1B). Interestingly, PDX101 and JQ-1 had contrasting effects on cleaved-PARP expression in the two resistant cell lines. In order to confirm this odd behavior, we measured the apoptosis using Annexin V/PI double staining. The results were generally consistent with the western blotting data (see Supplementary Fig. 2). UNC0638 treatment resulted in obvious apoptosis (Annexin V positive cells) in both resistant cell lines. In addition, PDX101 exposure induced apoptosis in PC9/ER cells but relatively weak in HCC827/ER cells, and JQ-1 treatment triggered the enhanced apoptosis in HCC827/ER cells but had no effect on PC9/ER cells, which suggested that the ability of these inhibitors to induce apoptosis is cell type dependent. Taken together, the above results indicated that several epigenetic enzymes, especially EHMT2, were associated with the inhibition of cell growth in NSCLC/ER cells.

### Upregulation of EHMT2 expression induced TKI resistance and the malignant phenotype in EGFR-TKI-resistant NSCLC cells

**Fig. 2:**
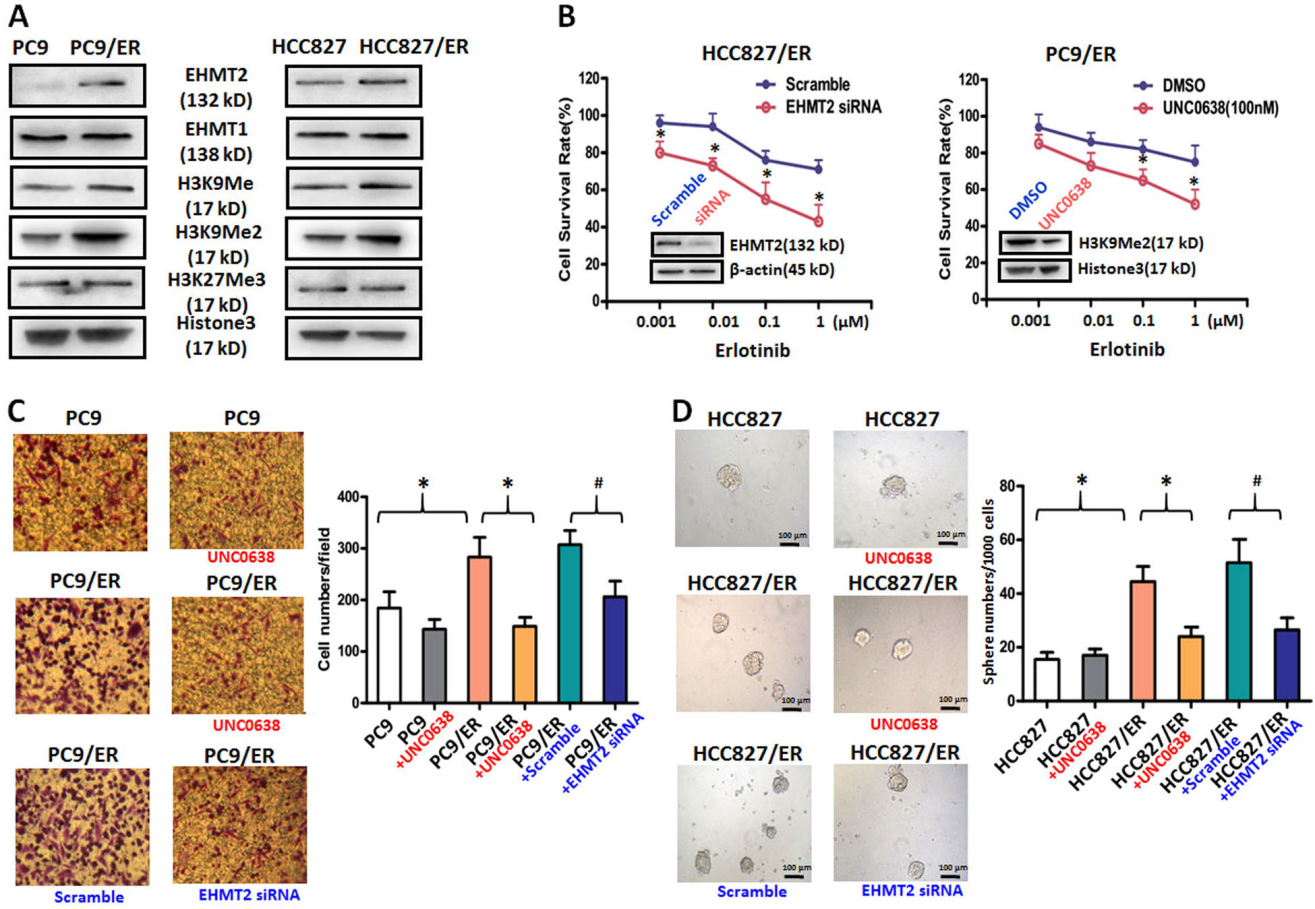
Effects of genetic or chemical manipulation of EHMT2 on the biological characteristics of EGFR-TKI-resistant NSCLC cells **a** EHMT2, EHMT1, H3K9Me, H3K9Me2, and H3K27Me3 expression levels were measured in EGFR-TKI-resistant and parental NSCLC cell lines. Histone-3 was used as a loading control. **b** Cell viability was measured in EHMT2-knockdown or UNC0638-treated PC9/ER cells treated with Erlotinib at different concentrations for 48 h. Scramble siRNA or DMSO was used as a control. **c** Cell migration was measured in PC9, PC9/ER and PC9/ER cells treated with 100 nM UNC0638 or 20 nM EHMT siRNA. Scramble siRNA or DMSO was used as a control. **d** Tumor sphere was counted in HCC827, HCC827/ER, and HCC827/ER cells treated with 100 nM UNC0638 or 20 nM EHMT siRNA. Scramble siRNA or DMSO was used as a control (scale bars, 100 μm). *P < 0.05, compared to corresponding control cells; ^**#**^*P* < 0.05, compared to resistant cells with Scramble siRNA treatment

To determine the role of EHMT2 in EGFR-TKI resistance in NSCLC cells, we measured the expression levels of EHMT2 and its partner molecule, EHMT1, in parental and resistant cells^12^. Our results showed that EHMT2 expression levels were upregulated in the PC9/ER and HCC827/ER cell lines compared to the corresponding parental cell lines (Fig. 2a). However, EHMT1 expression levels did not differ between the resistant and parental cell lines, suggesting that EHMT1 was not associated with EGFR-TKI resistance in NSCLC cells (Fig. 2a). Moreover, the expression levels of the EHMT2 histone targets H3K9Me and H3K9Me2, especially H3K9Me2, were increased in both resistant cell lines compared to the corresponding parental cell lines. On the contrary, the expression level of H3K27Me3, an EZH2 histone target, did not differ between the resistant and parental cell lines. Taken together, all of these results indicated that EHMT2 expression and activity levels were upregulated in EGFR-TKI-resistant cells.

To clarify the role of EHMT2 in EGFR-TKI-resistant NSCLC, we assessed the Erlotinib sensitivity of EGFR-TKI-resistant cells in which EHMT2 was inhibited. Our results demonstrated that inhibiting EHMT2 expression by EHMT2-specific siRNA transfection could enhance the sensitivity of PC9/ER and HCC827/ER cells to Erlotinib (Fig. 2b and Supplementary Fig. 3A). In contrast to EHMT2 knockdown, EHMT1 knockdown had no effect on the sensitivity of PC9/ER cells to Erlotinib (Supplementary Fig. 3B). Notably, PC9/ER cells pretreated with UNC0638 (100 nM) displayed enhanced sensitivity to Erlotinib compared to non-pretreated cells, suggesting that the inhibitor has value as a treatment for EGFR-TKI-resistant NSCLC (Fig. 2b). To confirm the crucial role of EHMT2 in the sensitivity to Erlotinib of PC9/ER cells, we further performed apoptosis analysis. As shown in Supplementary Fig 3C, the knockdown and inhibition of EHMT2 resulted in 1.63-folds (18.1% EHMT2 siRNA vs. 11.1% Scramble control) and 3.3-folds (44.0% UNC0638 vs. 13.3% DMSO control) increase at apoptosis cells, respectively. The above results demonstrated the important role of EHMT2 in EGFR-TKI resistance.

To comprehensively explore the role of EHMT2 in EGFR-TKI resistance, we next investigated the effects of the above treatments on cell migration and tumor sphere formation, phenomena that are considered characteristics of the malignant phenotype in EGFR-TKI-resistant cells^13,14^. Our results revealed that PC9/ER cells displayed enhanced migration ability compared to the corresponding parental cells and that treatment with inhibitor UNC0638 or knockdown by specific EHMT2 siRNA could suppress cell migration in PC9/ER cells (Fig. 2c). Similarly, the results of the tumor sphere formation assay indicated that treatment with UNC0638 or EHMT2 siRNA could reverse tumor resistance-mediated increases in the numbers of tumor spheres in HCC827/ER cells. Interestingly, UNC0638 exposure had no significant effect on malignant phenotype in parental cells (Fig. 2c and d), suggesting the unique role of EHMT2 in resistant cells. In summary, our results demonstrated that upregulating EHMT2 expression may be essential for the induction of EGFR-TKI resistance and the malignant phenotype in NSCLC.

### The AKT pathway was involved in EHMT2-mediated EGFR-TKI resistance in NSCLC

**Fig. 3:**
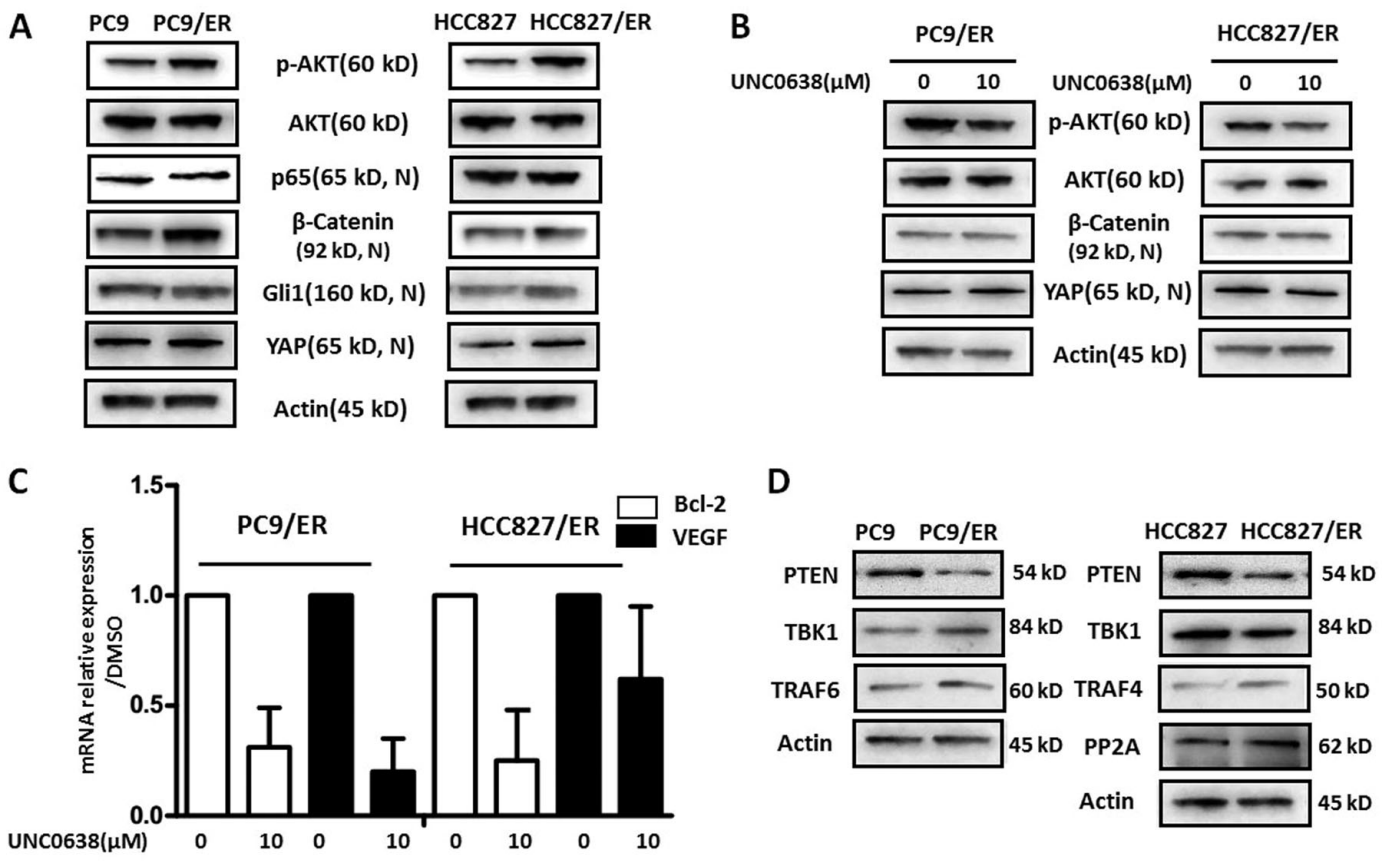
Feedback activation of TKI resistance-related signaling in EGFR-TKI-resistant NSCLC cells **a** p-AKT, AKT, nuclear p65, nuclear β-Catenin, nuclear Gli1, and nuclear YAP expression levels were measured in EGFR-TKI-resistant and parental NSCLC cell lines. β-actin was used as a loading control. **b** p-AKT, AKT, nuclear β-Catenin, and nuclear YAP expression levels were detected in EGFR-TKI-resistant cells treated with or without UNC0638. β-actin was used as a loading control. **c** Bcl-2 and VEGF mRNA expression levels were assessed by real-time RT-PCR in EGFR-TKI-resistant cells treated with or without UNC0638. **d** PTEN, TBK1, TRAF6, TRAF4, and PP2A expression levels were measured in EGFR-TKI-resistant and parental NSCLC cells. β-actin was used as a loading control

As epigenetic regulation is usually not involved in gene mutation and amplification, which are regarded as the main causes of EGFR-TKI resistance in NSCLC^5^, we presumed that activation of bypass pathways, including the AKT, NF-κB, WNT, Hedgehog, and YAP pathways, which were reported to affect EGFR-TKI resistance by self-activation^15–19^, may be associated with EHMT2-mediated EGFR-TKI resistance in NSCLC cells. We subsequently assessed the expression levels of several key molecules in those pathways in both resistant and parental cells. As shown in Fig. 3a and Supplementary Fig. 4, phosphorylated AKT (p-AKT) expression levels but not total AKT expression levels were upregulated in both resistant cell lines compared to the corresponding parental cell lines. Additionally, nuclear expression of β-Catenin, a transcription factor mediating the WNT pathway, was increased in PC9/ER cells but not in HCC827 cells compared to the corresponding parental cells. YAP, a crucial protein in Hippo/YAP signaling, was highly expressed in HCC827/ER cells compared to the corresponding parental cells. However, we noted no significant differences in the expression levels of p65 and Gli1(Fig. 3a and Supplementary Fig. 4), which are a biomarker of NF-κB and a key transcription factor in the Hedgehog pathway, respectively, between the resistant and parental cell lines. To clarify the roles of these pathways in EHMT2-mediated EGFR-TKI resistance, we determined p-AKT, AKT, β-Catenin, YAP, and actin expression levels in the indicated resistant NSCLC cell lines, which were treated with or without UNC0638. The results indicated that p-AKT expression levels were reduced in both treated cell lines compared to both untreated cell lines; however, β-Catenin and YAP expression levels did not differ between the treated and untreated cell lines (Fig. 3b). We also verified the role of the AKT pathway in EHMT2-mediated EGFR-TKI resistance by assessing the expression levels of the genes encoding AKT pathway-related proteins. We found that treatment with UNC0638 resulted in the downregulation of Bcl-2 and VEGF in the corresponding cells compared to untreated cells (Fig. 3c). The above data suggested that AKT pathway activation may be associated with EHMT2-mediated EGFR-TKI resistance.

To elucidate the mechanism by which the AKT pathway regulates EHMT2-mediated EGFR-TKI resistance, we detected the expression levels of several molecules upstream of the AKT pathway, including PTEN, TBK1, TRAF4, TRAF6 and PP2A, in the resistant and parental cell lines. As shown in Fig. 3d, PTEN expression levels were reduced in the resistant cell lines compared to the parental cell lines, whereas the other proteins upstream of the AKT pathway were not decreased in the resistant cell lines compared to the parental cell lines, indicating that insufficient PTEN expression contributes to AKT activation in EGFR-TKI-resistant NSCLC cells. All of these findings suggested that PTEN-mediated AKT pathway activation may be associated with EHMT2-mediated EGFR-TKI resistance in NSCLC cells.

### EHMT2 epigenetically regulated PTEN expression in EGFR-TKI-resistant NSCLC cells

**Fig. 4:**
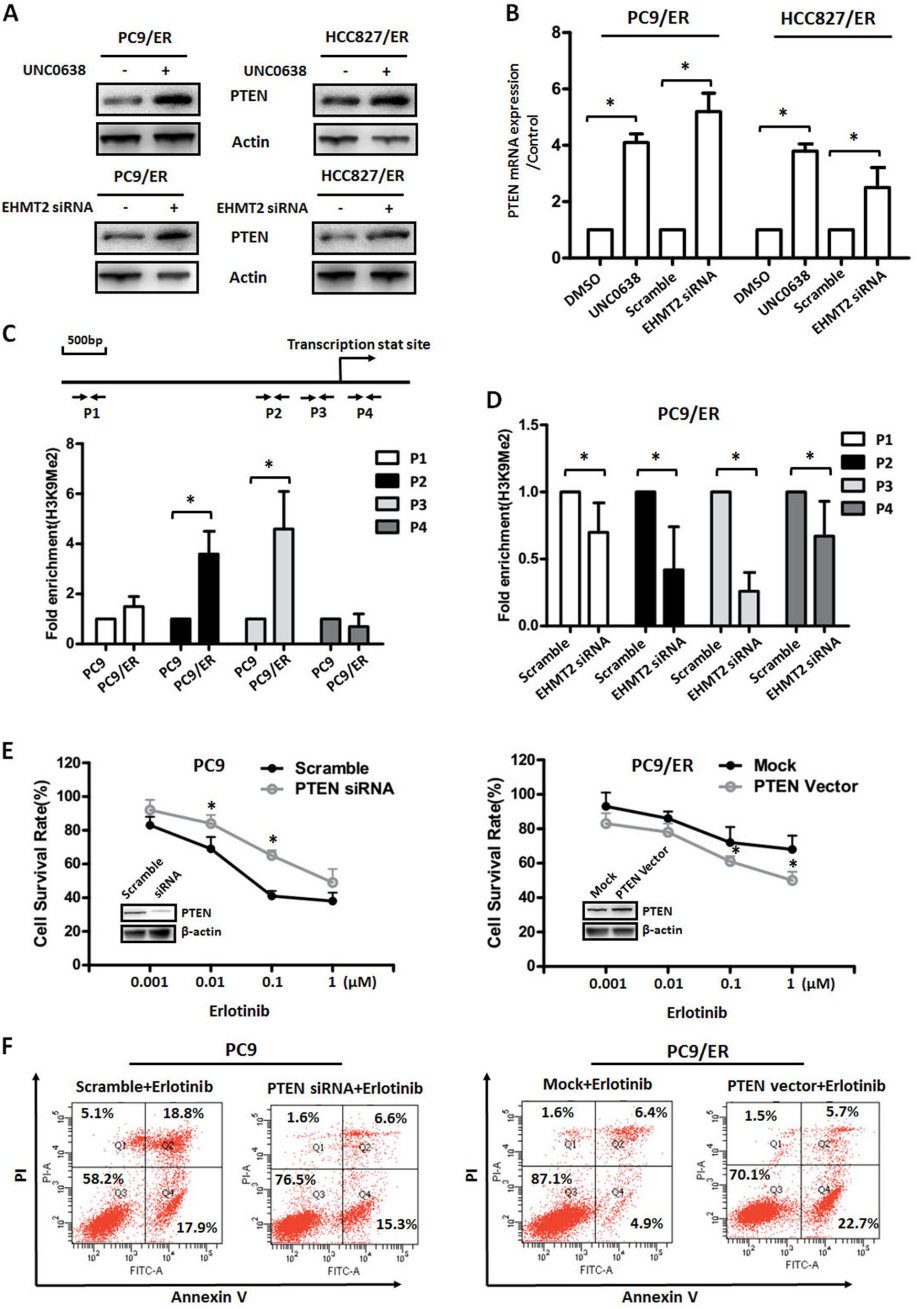
Epigenetic regulation of PTEN and its role in EGFR-TKI resistance in NSCLC **a** PTEN expression was detected in EGFR-TKI-resistant cells treated with 10 μM UNC0638 or transfected with 20 nM EHMT2-specific siRNA. β-actin was used as a loading control. **b** PTEN mRNA expression levels were assessed by real-time RT-PCR in EGFR-TKI-resistant cells treated with 10 μM UNC0638 or transfected with 20 nM EHMT2-specific siRNA. GAPDH was used as a control. **c** ChIP assays were performed to measure the ability of H3K9Me2 to bind different promoter and enhancer regions in the *PTEN* gene (P1, P2, P3, and P4) in PC9 and PC9/ER cells. **d** H3K9Me2 enrichment was accessed in PC9/ER cells with or without EHMT2 inhibition. **e** Cell viability in PC9 or PC9/ER cells, with PTEN gene manipulation, which were treated with Erlotinib at different concentrations for 48 h. Scramble siRNA or mock vector was used as a control. **f** Cell apoptosis was assessed using Annexin V/PI double staining in *PTEN* gene manipulated PC9 or PC9/ER cells which were treated with Erlotinib at designed concentrations (0.1 μM for PC9 cells and 1 μM for PC9/ER cells) for 48 h. Scramble siRNA or mock vector was used as a control. * *P* < 0.05, compared to corresponding control cells

Activation of EHMT2, which plays a role in transcriptional inhibition^20^, is accompanied by downregulation of PTEN in EGFR-TKI-resistant cells, suggesting that EHMT2 is linked to the epigenetic regulation of PTEN expression. To confirm this hypothesis, we first examined PTEN protein expression levels in both resistant cell lines, in which EHMT2 was or was not manipulated. Our results indicated that either treatment with UNC0638 or siRNA-mediated inhibition of EHMT2 expression led to the upregulation of PTEN expression in both resistant cell lines compared to the corresponding control cell lines (Fig. 4a). To confirm whether PTEN upregulation was caused by transcriptional regulation, we measured PTEN mRNA expression levels in both resistant cell lines, which were treated with or without UNC0638 or EHMT2-specific siRNA. As shown in Fig. 4b, treatment with UNC0638 and EHMT2 siRNA resulted in significant upregulation of PTEN mRNA expression levels (2.5–5 folds) in the corresponding groups compared to the control groups (DMSO and Scramble groups), suggesting that EHMT2 regulates PTEN expression at the transcriptional level in EGFR-TKI-resistant NSCLC cells.

To confirm whether direct epigenetic regulation of PTEN expression was involved in EGFR-TKI resistance in NSCLC cells, we measured the binding ability of H3K9Me2 and H3K9Ac, markers of active gene expression, in PC9/ER and parental cells. Our results demonstrated that H3K9Me2 bound the P2 and P3 regions of the PTEN gene promoter to a greater extent in PC9/ER cells than in PC9 cells (Fig. 4c). However, H3K9Ac bound the P2 and P3 regions of the PTEN gene promoter to a lesser extent in PC9/ER cells than in PC9 cells (Supplementary Fig. 5A). Furthermore, inhibiting EHMT2 expression contributed to decreases in the extent to which H3K9Me2 was enriched in the indicated four regions (P1, P2, P3, and P4), especially the P2 and P3 regions (Fig. 4d), suggesting that EHMT2 plays a role in PTEN transcriptional regulation in EGFR-TKI-resistant cells. Similarly, knocking down EHTM2 expression with EHTM2-specific siRNA transfection also altered the extent to which H3K9Ac was enriched in the P2 and P3 regions in PC9/ER cells (Supplementary Fig. 5B). The above results demonstrated that EHMT2 epigenetically regulates PTEN expression in EGFR-TKI-resistant NSCLC cells.

To clarify the role of PTEN in EGFR-TKI resistance, we next detected the effects of *PTEN* gene manipulation on the sensitivity of NSCLC cells to EGFR-TKI. Our results showed that knocking down PTEN expression in PC9 cells contributed to the resistance of PC9 cells to Erlotinib, whereas overexpressing PTEN in PC9/ER cells increased the sensitivity of the cells to Erlotinib (Fig. 4e). Furthermore, the Annexin V/PI double staining results confirmed that gene manipulation of *PTEN* affected apoptosis induced by Erlotinib in PC9 and PC9/ER cells (Fig. 4f). The above data indicating that PTEN expression regulation was associated with EGFR-TKI resistance in NSCLC.

### Targeting EHMT2 enhanced the antitumor effects of Erlotinib in EGFR-TKI-resistant xenograft models

**Fig. 5:**
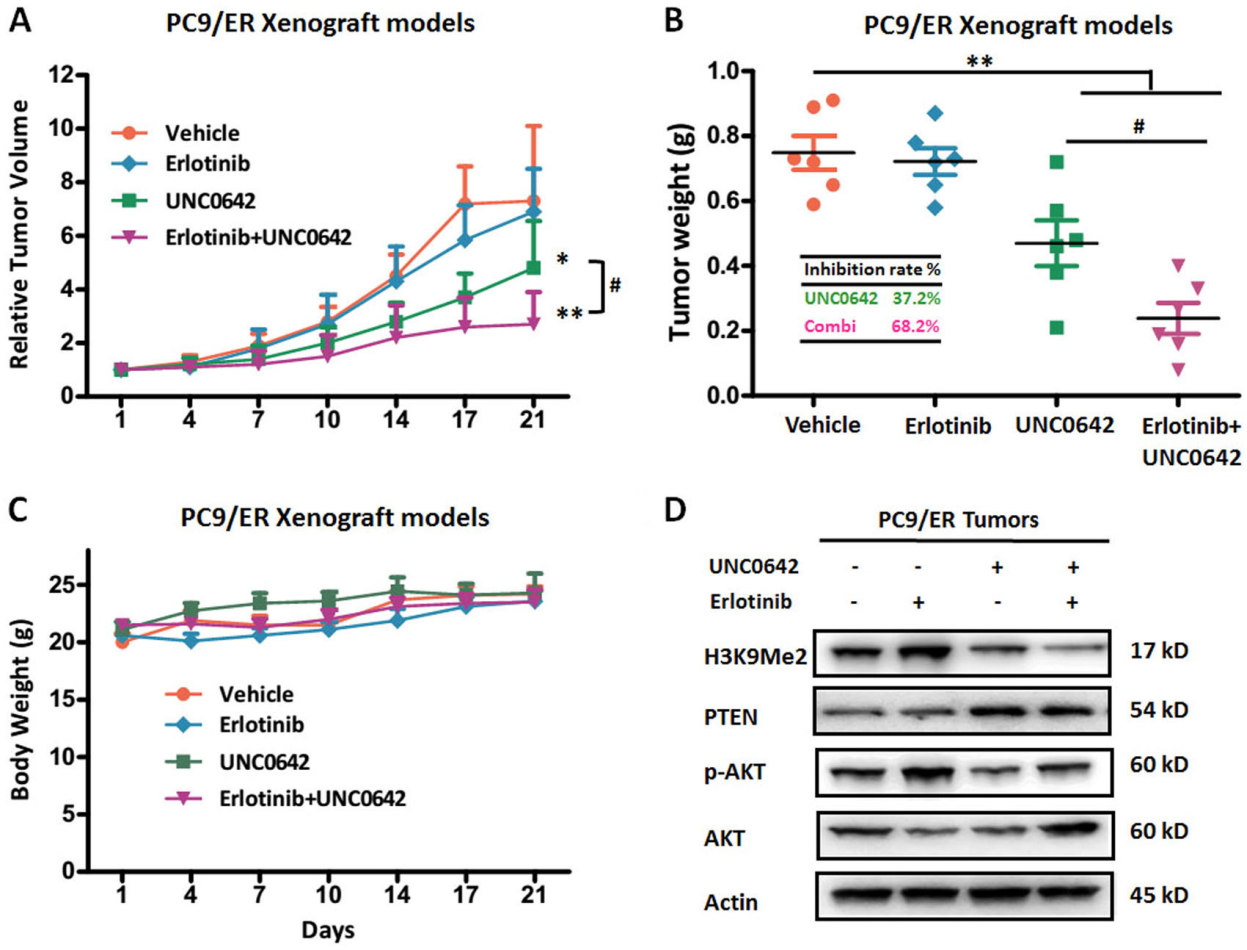
Effects of EHMT2 inhibiton and/or Erlotinib on tumor growth in an EGFR-TKI-resistant xenograft model **a** Relative tumor volumes, **b** tumor weights, and **c** body weights were measured in PC9/ER xenografts treated with UNC0642, Erlotinib or the combination of UNC0642 and Erlotinib. **d** H3K9Me2, PTEN, p-AKT, and AKT expression levels were measured in PC9/ER xenograft tumor tissues. β-actin was used as a loading control. *P < 0.05, **P < 0.01, compared to control; ^**#**^*P* < 0.05, combined treatment group compared to UNC0642 single treatment group

As our previous results demonstrated that EHMT2 expression and activity levels were increased in EGFR-TKI-resistant NSCLC cells (Fig. 2a), we performed additional investigations to confirm whether ablation of EHMT2 expression could sensitize resistant NSCLC cells to TKI in vivo. We treated SCID mice bearing PC9/ER xenografts with or without Erlotinib and/or UNC0642 (in vivo EHMT2 inhibitor) after the tumors reached an average volume of 80 mm^3^. Our results indicated that the tumor growth was not inhibited in the Erlotinib-treated group compared to the control group (Fig. 5a, b), a result consistent with those of the experiments involving the PC9/ER cell line (Fig. 2b). Furthermore, our results showed that UNC0642 administration led to moderate suppression of tumor growth in the corresponding group of mice compared to the control group of mice. The tumor inhibition rate was 37.2% in the former group of mice (Fig. 5b). Notably, PC9/ER xenograft mice treated with both agents displayed a significant inhibition, with tumor growth inhibition rate of 68.2%, as compared to that in single treatment group (*P* < 0.05, Fig. 5a, b). Additionally, all four groups of mice showed stable body weights, indicating that treatment with Erlotinib or UNC0642 was not associated with significant toxicity (Fig. 5c). Furthermore, we also examined H3K9Me2, PTEN and p-AKT expression levels in tumor tissue samples from the four groups (Fig. 5d). Consistent with our in vitro results, these results showed that administration of UNC0642 could reduce H3K9Me2 expression and increase PTEN expression and thus inhibit AKT phosphorylation. Treatment with Erlotinib alone induced H3K9Me2 upregulation and AKT pathway activation. However, treatment with the combination of the two drugs resulted in a reduction in H3K9Me2 expression and inhibition of the AKT pathway. These findings demonstrated that ablation of EHMT2 could sensitize EGFR-TKI-resistant cells in vivo by inhibiting the AKT pathway.

### High EHMT2 expression and low PTEN expression are predictive of a poor prognosis in NSCLC

**Fig. 6:**
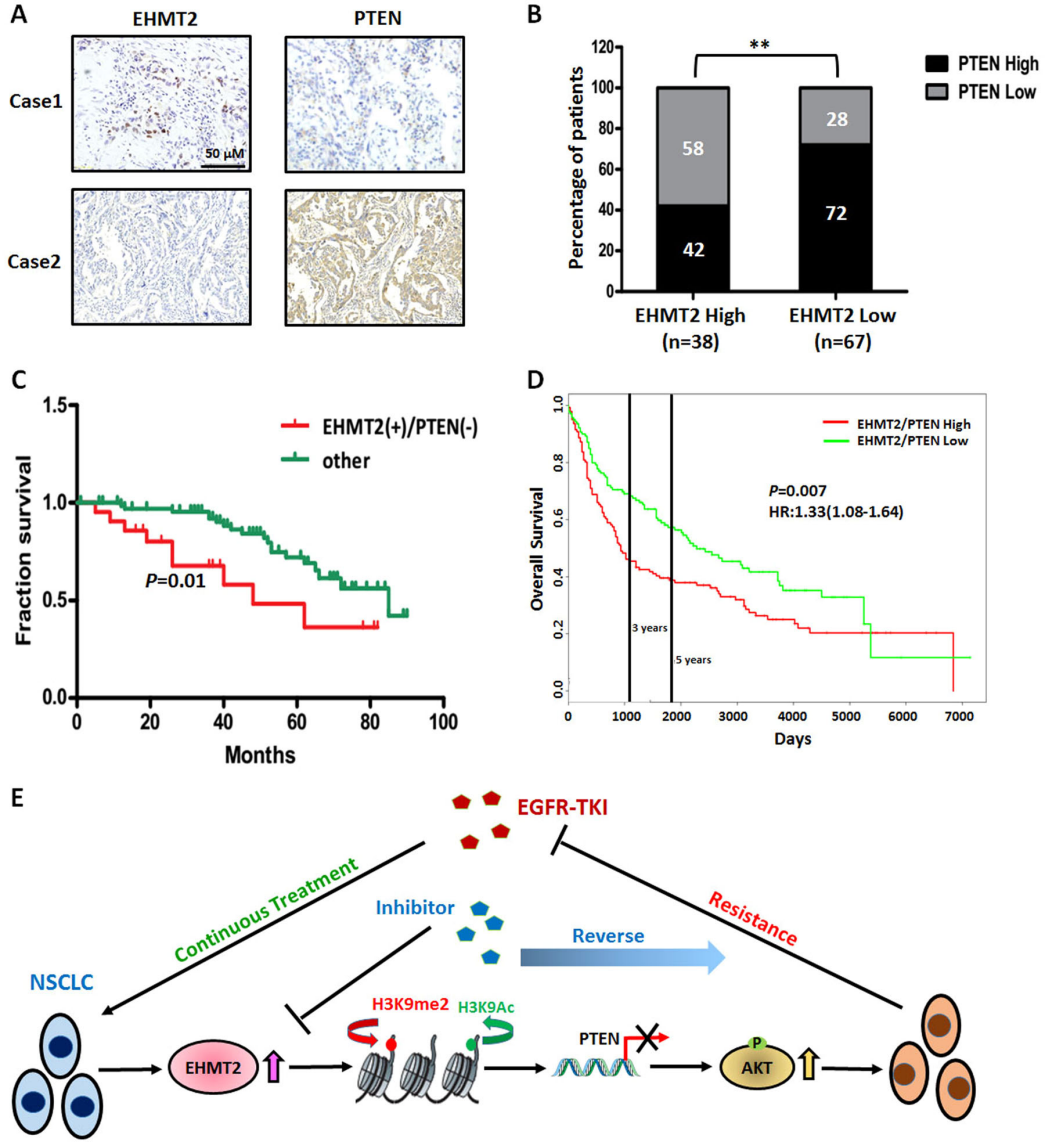
Measurement of EHMT2 and PTEN expression in clinical NSCLC specimens **a** Representative images of EHMT2 and PTEN expression in NSCLC tissues. **b** The correlation between EHMT2 expression and PTEN expression in NSCLC tissue specimens. **c** The combination of high EHMT2 expression and low PTEN expression was correlated with a poor prognosis in patients with NSCLC. **d** Overall survival rates according to data from the PROGgeneV2 database. **e** Schematic of the mechanism by which EHMT2 triggers the AKT pathway and induces EGFR-TKI resistance by epigenetically regulating PTEN expression in NSCLCs

We next investigated whether the above-mentioned relationship between EHMT2 and PTEN was present in human NSCLC tissues. We performed immunohistochemistry to determine EHTM2 and PTEN expression levels in paraffin-embedded tissue specimens from 105 patients with NSCLC (Fig. 6a). We found that 58% of cases with lower PTEN expression (*n* = 22) were in the high EHMT2 expression group (*n* = 38), whereas 72% of cases with higher PTEN expression (*n* = 48) were in the low EHMT2 expression group (*n* = 67), indicating that EHMT2 expression was negatively correlated with PTEN expression in NSCLC tissues (*P* < 0.05, Fig. 6b). Notably, prognostic analysis demonstrated that this expression pattern (higher EHMT2 expression and lower PTEN expression) was correlated with poorer overall survival than other expression patterns in patients with NSCLC (Fig. 6c), data consistent with those in the PROGgeneV2 database (GSE30219, Fig. 6d). Taken together, our results showed EHMT2 expression is negatively correlated with PTEN expression and that the balance between EHMT2 and PTEN expression levels may serve as a predictive marker for the prognoses of patients with NSCLC.

## Discussion

EGFR-TKIs are widely used against NSCLC with EGFR mutations; thus, preventing EGFR-TKI resistance is a crucial clinical issue with respect to the treatment of NSCLC with EGFR mutations^6^. In the present study, we found that EHMT2 plays an important role in EGFR-TKI resistance in NSCLC by epigenetically regulating various factors in EGFR-TKI-resistant cells. Our results demonstrated that suppression of EHMT2 sensitized resistant cells to Erlotinib. Regarding the mechanism by which EHMT2 facilitates treatment resistance, we found that EHMT2 can activate the AKT pathway by epigenetically regulating PTEN expression. Importantly, the combination of the above-mentioned EHMT2 inhibitor and Erlotinib had enhanced antitumor effects in EGFR-TKI-resistant NSCLC xenograft models. Clinical prognostic analysis showed that tumors with higher EHMT2 expression along with lower PTEN expression may have a poorer prognosis than tumors with other expression patterns in patients with NSCLC. Our findings provide us with a novel perspective regarding the epigenetic phenomena underlying EGFR-TKI resistance and indicate that the above agents may have promise as treatments for patients with EGFR-TKI-resistant NSCLC.

EHMT2, also known as G9a, is a histone methyltransferase that catalyzes the monomethylation and dimethylation of H3K9^12^, which has been characterized as a transcriptional coactivator and corepressor^21,22^. Several reports have indicated that EHMT2 expression and enzymatic activity were associated with malignant behaviors, such as tumor growth, cell invasion, cancer stem cell phenotypes and survival, in various cancer types^23–25^. Liu et al.^26^ recently found that EHMT2 protected head and neck squamous cell carcinoma cells against cisplatin by increasing GSH expression, suggesting that EHMT2 is a promising target for the prevention of chemotherapy resistance in patients undergoing cancer treatment. Our results also demonstrated that EHMT2 expression and enzymatic activity levels were upregulated in NSCLC EGFR-TKI-resistant cells. Moreover, either knocking down EHMT2 expression or inhibiting EHMT2 activity resulted in increases in sensitivity to treatment with TKIs and decreases in cell migration and self-renewal ability in resistant NSCLC cells, suggesting that EHMT2 plays an important role in EGFR-TKI resistance in NSCLC (Fig. 2).

As an epigenetic enzyme, EHMT2 executes its function mainly by regulating the expression of certain target genes rather than by inducing genetic modifications, such as mutations or amplifications, which are usually considered the primary causes of EGFR-TKI resistance^5,12^. After assessing its involvement in signaling pathways and measuring the expression of its upstream molecules, we determined that EHMT2 participated in EGFR-TKI resistance by regulating the PTEN/AKT pathway in NSCLC (Fig. 3). We found that EHMT2 epigenetically promotes H3K9Me2 enrichment and dynamically contributes to decreases in H3K9Ac enrichment in the *PTEN* gene promoter region, resulting in transcriptional repression of the *PTEN* gene (Fig. 4 and Supplementary Fig. 2), which leads to the relief of AKT pathway inhibition and, eventually, to AKT pathway activation. Several studies have demonstrated that PTEN expression was regulated by methylation of CpG islands in the promoter region in various types of cancer^27,28^. A previous study showed that histone acetylation status was associated with the regulation of PTEN expression^29^. Our results also showed that changes in histone methylation, especially changes in H3K9 methylation, contribute to the regulation of PTEN expression in NSCLC. Taken together, these findings indicate that epigenetic regulation plays a crucial role in PTEN expression and is involved in processes associated with malignant transformation, such as drug resistance. However, the relationship between DNA methylation and histone modification, as well as the roles of these processes in PTEN regulation, needs to be studied further.

Inevitable resistance to TKIs in NSCLC usually results in treatment failure. However, our observations regarding the effects of EHMT2 in EGFR-TKI-resistant cells and our finding that EHMT2 positively regulates the AKT pathway, prompted us to evaluate the effectiveness of combination therapy as a treatment for EGFR-TKI-resistant NSCLC. Our findings indicated that administering an EHMT2 inhibitor in combination with Erlotinib had a significant effect on tumor growth in preclinical models. Notably, a recent study showed that an inhibitor of the protein methyltransferase EZH2 could eradicate TKI resistance in chronic myelogenous leukemia^30^. Given that EHMT2 has reversible effects and is a targetable protein methyltransferase, treatments targeting the protein may be useful for reversing TKI resistance in NSCLC.

In conclusion, the present study demonstrated that elevated EHMT2 expression and activity in NSCLC may induce resistance to treatment with EGFR-TKI, indicating that EHMT2 may be a useful indicator of EGFR-TKI resistance in NSCLC. Upregulation of EHMT2 expression results in the epigenetic-mediated downregulation of PTEN expression and thus contributes to AKT pathway activation (Fig. 6e). Moreover, combination treatment with an EHMT2 inhibitor and Erlotinib had enhanced antitumor effects in a xenograft model in vivo. Therefore, we not only illustrated that epigenetic phenomena are responsible for EGFR-TKI resistance in NSCLC but also showed that the abovementioned novel therapeutic strategy may be useful for NSCLCs.

## Materials and methods

### Cell lines, cell culture, and treatment

PC9 and HCC827 human lung adenocarcinoma cell lines with an EGFR-activating mutation (deletion in exon 19) were purchased from Immuno-Biological Laboratories (Fujioka, Japan) and the American Type Culture Collection (ATCC; Manassas, VA, USA), respectively. These cancer cells were routinely cultured in RPMI-1640 medium (Gibco, Grand Island, NY, USA) supplemented with 10% fetal bovine serum (FBS; Gibco) and were maintained at 37 °C in a humidified incubator with 5% CO_2_. The cells were treated with Erlotinib (J&K Scientific Ltd., Beijing, China) at increasing concentrations (ranging from 0.5 to 2 μM) for 3 months.

### Compounds and reagents

5-Azacytidine (5-AZA), PXD101, JQ-1, UNC0638, UNC0642, EPZ5676, GSK-JQ, GSK-126, UNC0379, and LLY-507 were obtained from MedChem Express (Princeton, NJ, USA). The primary antibodies against Cleaved-PARP, H3K9Me, H3K9Me2, H3K27Me3, H3K9Ac, Histone3, Oct4, Akt, phosphor-Akt, NF-κB p65, β-Catenin, Gli1, YAP, TBK1, TRAF4, PP2A and β-actin were obtained from Cell Signaling Technology (Danvers, MA, USA), and the primary antibodies against EHMT2 and EHMT1 were purchased from Abcam (Cambridge, UK). The pcDNA3-PTEN vector was generated in our laboratory.

### Patients and therapy

A total of 105 patients with NSCLC (stage I–stage III) who were treated at Wuhan General Hospital (Wuhan, Hubei Province, China) were enrolled in the study between January 2004 and June 2012. The clinical characteristics of these patients are presented in Supplementary Table S1. Ethical approval was obtained from the Institutional Review Board of Wuhan General Hospital.

### Immunohistochemistry

A tissue microarray was constructed (in collaboration with Shanghai Biochip Company Ltd., Shanghai, China) as described previously^31^. Paraffin-embedded tissue sections were dewaxed with xylene and rehydrated in descending concentrations of ethanol. Endogenous peroxidase activity was inhibited, and then the slides were incubated with antibodies against EHMT2 (1:50; Abcam, Cambridge, England) and PTEN (1:100; Cell Signaling Technology, Danvers, MA, USA). EHMT2 and PTEN expression levels were assessed as described in Lee et al.^32^

### Cell viability assay

In vitro cell viability was determined using the MTT assay. Cells (8 × 10^4^ cells/ml) were seeded in 96-well culture plates. After incubating overnight, the cells were treated with various concentrations of the appropriate agents for 48 h, after which 10 μl of MTT solution (2.5 mg/ml in PBS) was added to each well, and the plates were incubated for an additional 4 h at 37 °C. After the samples were centrifuged (2500 rpm, 10 min), the medium supplemented with MTT was aspirated, and then 100 μl of DMSO was added to each well. The optical density of each well was measured at 570 nm with a Biotek SynergyTM HT Reader (BioTek Instruments, Winooski, VT, USA).

### Western blot analysis

Western blotting was performed as previously described^33^. Briefly, equal amounts of total protein extracts from cultured cells or tissues were fractionated by 10–15% SDS-PAGE before being electrically transferred onto polyvinylidene difluoride membranes, which were sequentially incubated with mouse or rabbit primary antibodies and horseradish peroxidase-conjugated secondary antibodies designed to detect the proteins of interest. The indicated secondary antibodies were subsequently reacted with ECL detection reagents (Pierce, Thermo Fisher Scientific, Waltham, MA, USA) and then incubated in a dark room. The relative expression levels of the indicated proteins were normalized to those of β-actin or Histone3, each of which served as an internal control.

### Flow cytometry analysis

Analyses for apoptosis were conducted with an Annexin V–FITC Apoptosis Detection Kit (BioVision, Mountain View, CA, USA). Cells (1 × 10^6^) were exposed to various inhibitors for 48 h. They were collected by centrifugation and resuspended in 500 μl of 1× binding buffer. Annexin V–fluorescein isothiocyanate (FITC; 5 μl) and PI (5 μl) were added to the cells. After incubation at room temperature for 5 min in the dark, cells were analyzed by FACS using a flow cytometer (BD Biosciences, San Jose, CA, USA). Cells that stained Annexin V–FITC (apoptosis) were analyzed.

### siRNA-mediated gene knockdown

*EHMT2* and *EHMT1* knockdown was performed using specific siRNAs purchased from Santa Cruz Biotechnology (Santa Cruz Biotechnology, Santa Cruz, CA, USA). Scramble non-target siRNAs served as negative controls. siRNA was introduced into the indicated cell lines with Lipofectamine RNAiMAX reagent (Thermo Fisher Scientific), according to the manufacturer’s instructions, and knockdown efficiency was assessed by western blotting.

### Transwell migration assay

PC9 and PC9/ER migration capacity was tested by Corning transwell assay, according to the manufacturer’s instructions. Briefly, the indicated lung cancer cells were treated with DMSO, UNC0638 (100 nM), Scramble siRNA, and EHMT2 siRNA(20 nM) for 48 h and then seeded in the upper chamber of the system at a density of 5 × 10^4^ cells/well in serum-free medium (100 μl). The wells in the lower chamber of the system were filled with complete medium. After incubating for 48 h, the cells remaining in the upper chamber were carefully removed with a cotton swab, and the cells that had migrated through the membrane and adhered to its lower surface were fixed with 100% methanol and stained with 0.2% crystal violet. The membrane was then photographed under a microscope, and the cells in five predetermined fields were counted at 200× magnification.

### Tumor sphere formation assay

HCC827 and HCC827/ER cells were treated with DMSO, UNC0638 (100 nM), Scramble siRNA, and EHMT2 siRNA (20 nM) for 48 h, after which single cells prepared by mechanical and enzymatic dissociation were seeded in six-well ultra-low attachment plates (Corning, NY, USA) at a density of 1000 cells/well in serum-free DMEM/F-12 medium supplemented with B27 (1×, Invitrogen, Thermo Fisher Scientific), 20 ng/ml human recombinant bFGF (PeproTech, Rocky Hill, NJ, USA), and 20 ng/ml EGF (PeproTech) for 10–14 days. The cells were then photographed under a microscope.

### Quantitative PCR analysis

Total RNA was isolated using an RNeasy Mini Kit (Qiagen, Hilden, Germany), as described in the product insert, and then reverse transcribed with a RevertAid First Strand cDNA Synthesis Kit (Thermo Fisher Scientific). PCR was performed with iQ SYBR Green SuperMix (Bio-Red Laboratories, Hercules, CA, USA) and a CFX96 Real-Time PCR Detection System (Bio-Rad Laboratories). The following primers were used for the experiment: glyceraldehyde-3-phosphate dehydrogenase (*GAPDH*): reverse: 5′-CCCTCAACGACCACTTTGTCA-3′ and forward: 5′-TTCCTCTTGTGCTCTTGCTGG-3′; *Bcl-2*: reverse: 5′-CAGCCAGGAGAAATCAAACAGAGG-3′ and forward: 5′-ATCGCCCTG TGGATGACTGAG-3′; *VEGF*: reverse: 5′-TGAATTCTCAGCCCTCTTCAA-3′ and forward: 5′-TCTGCAGCTCTGTGTGAAGG-3′; and *PTEN* reverse: 5′-AACTGGCAGGTAGAAGGCAACTC-3′ and forward: 5′-CGGCAGCATCAAATGTTTCAG-3′.

### Chromatin immuno-precipitation assay (ChIP)

The ChIP Assay Kit was purchased from Beyotime Biotechnology (Shanghai, China). PC9 and PC9/ER cells were prepared for ChIP assay, which was performed according to the instructions of the manufacturer. H3K9Me2 and H3K9Ac antibodies were used for immunoprecipitation. PTEN promoter primers were used to amplify the DNA isolated by ChIP assay by PCR, and real-time PCR was performed to analyze the amplification product. The following PCR primers were used for the experiments: P1 forward: 5′-CGGTGGCTCACGCCTGTAAT-3′ and reverse: 5′-CACTGCAACCTCTGCCTCCC-3′; P2 forward: 5′-GCAGGAAGGGTTGGGGTTCC-3′ and reverse: 5′-GGATACACGGGCCACAGTCG-3′; P3 forward: 5′-GAGCCCGAGGGGAAAGATGC-3′ and reverse: 5′-AAAGCTCTCAGCCGAGCGTG-3′; and P4 forward: 5′-TCATCAGTCCTCCACCCCCG-3′ and reverse: 5′-TGCACTTGCTGCGGCTTTTG-3′.

### Xenografts in mice

To assess the characteristics of chemotherapy-resistant tumors, we subcutaneously injected viable PC9/RE cells (5 × 10^6^/100 μl PBS per mouse), as confirmed by trypan blue staining, into the right flank of 7–8-week-old male SCID mice. When the average tumor volume reached 50 mm^3^, the mice were randomly divided into the following four treatment groups: a control group (saline only, *n* = 6), a UNC0642 group (2.5 mg/kg/bid, i.p.; *n* = 6), an Erlotinib group (12.5 mg/kg/day, i.p.; *n* = 6), and a combination treatment group (UNC0642 plus Erlotinib). After 3 weeks, the mice were sacrificed, and the tumors were excised and stored at −80 °C. These experiments were performed in strict accordance with the recommendations in the Guide for the Care and Use of Laboratory Animals of the National Institutes of Health, and the corresponding protocol was approved by the Animal Experimental Ethics Committee of Shenyang Pharmaceutical University (Shenyang, Liaoning Province, China).

### Statistical analysis

Differences between the indicated experimental groups were evaluated by one-way ANOVA or Turkey’s post hoc test with the SPSS 11.5 software package for Windows (SPSS, Chicago, IL, USA), and survival curves were constructed using the Kaplan–Meier method. *P*-values less than 0.05 were considered statistically significant (*P* < 0.05, two-tailed test).

## Electronic supplementary material

Supplementary table 1

Supplementary figure 1

Supplementary figure 2

Supplementary figure 3

Supplementary figure 4

Supplementary figure 5

Supplementary Figure Legends
