# Supplementary material for: Targeting EHMT2 reverses EGFR-TKI resistance in NSCLC by epigenetically regulating the PTEN/AKT signaling pathway
Source: Cell Death Dis. 2018 Jan 26;9(2):129. doi: 10.1038/s41419-017-0120-6 (PMC5833639; doi:10.1038/s41419-017-0120-6)
Supplement: Supplementary file 1 — Supplementary table 1 [file 41419_2017_120_MOESM1_ESM.docx]

**Supplementary table 1. Clinicopathological parameters in lung cancer patients．**

| **Variable** | **N** |
| --- | --- |
| **Age** |  |
| < 65 years | 47 |
| ≥ 65 years | 58 |
| **Tumor size** |  |
| < 3.0 cm | 43 |
| ≥ 3.0 cm | 62 |
| **Node metastasis** |  |
| Negative | 61 |
| Positive | 44 |
| **Histologic type** |  |
| Squamous carcinoma | 32 |
| Adenocarcinoma | 73 |
| **pTNM staging** |  |
| 0-I | 40 |
| II | 35 |
| III | 30 |
