# Supplementary figures and images for: Targeting EHMT2 reverses EGFR-TKI resistance in NSCLC by epigenetically regulating the PTEN/AKT signaling pathway
Source: Cell Death Dis. 2018 Jan 26;9(2):129. doi: 10.1038/s41419-017-0120-6 (PMC5833639; doi:10.1038/s41419-017-0120-6)

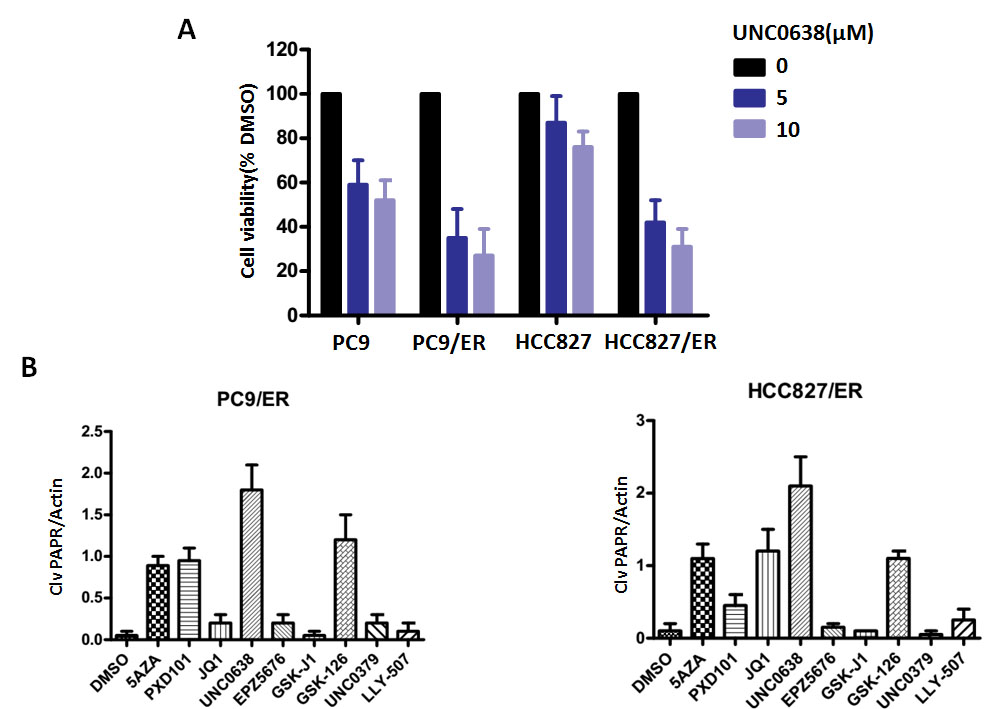

Supplement: Supplementary file 2 — Supplementary figure 1 [file 41419_2017_120_MOESM2_ESM.jpg]

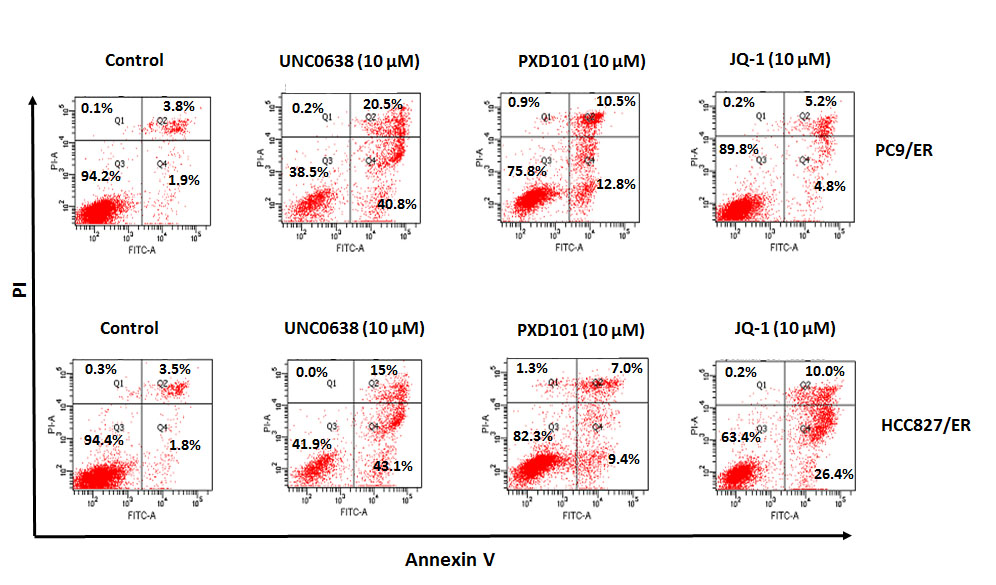

Supplement: Supplementary file 3 — Supplementary figure 2 [file 41419_2017_120_MOESM3_ESM.jpg]

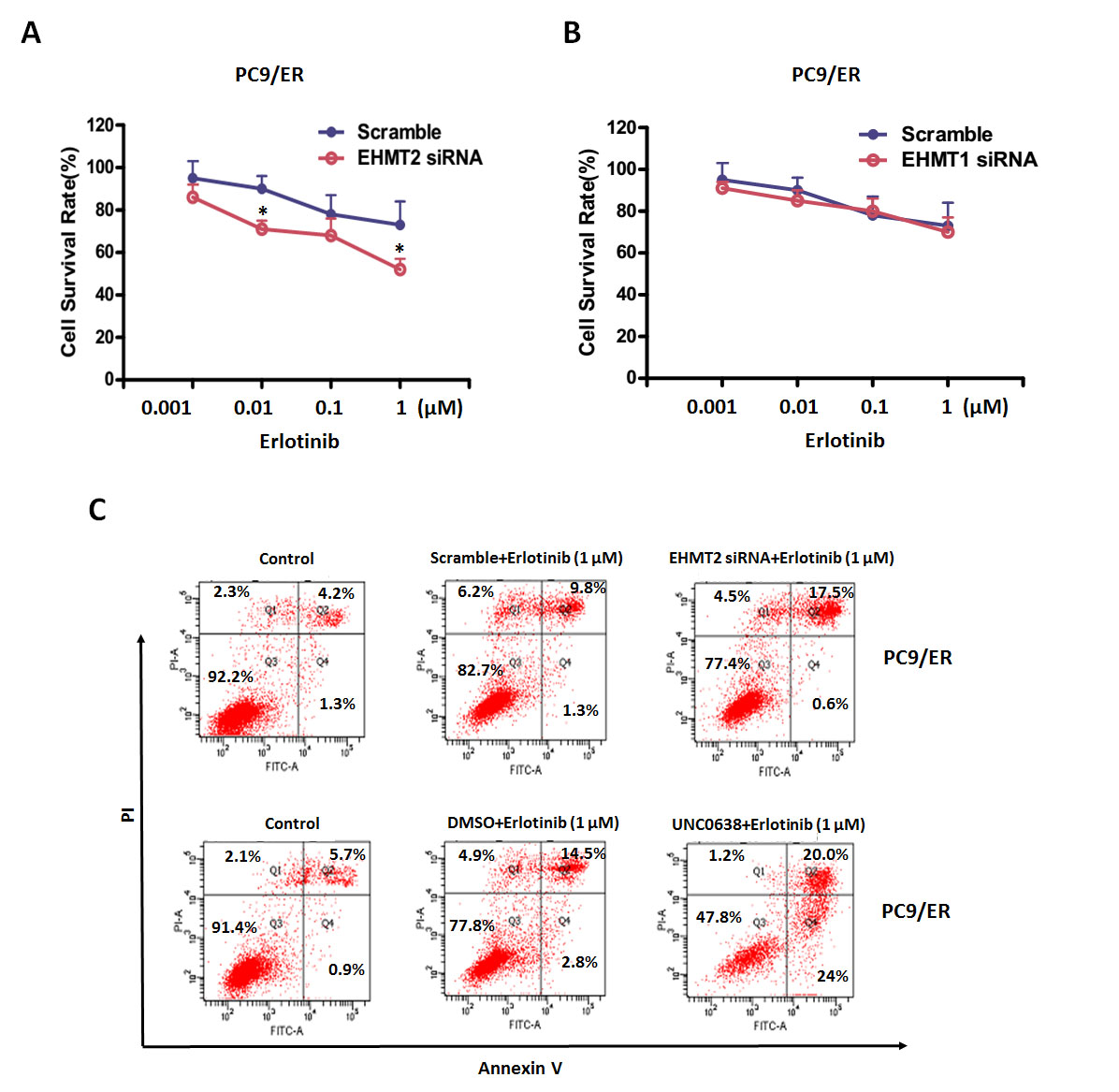

Supplement: Supplementary file 4 — Supplementary figure 3 [file 41419_2017_120_MOESM4_ESM.jpg]

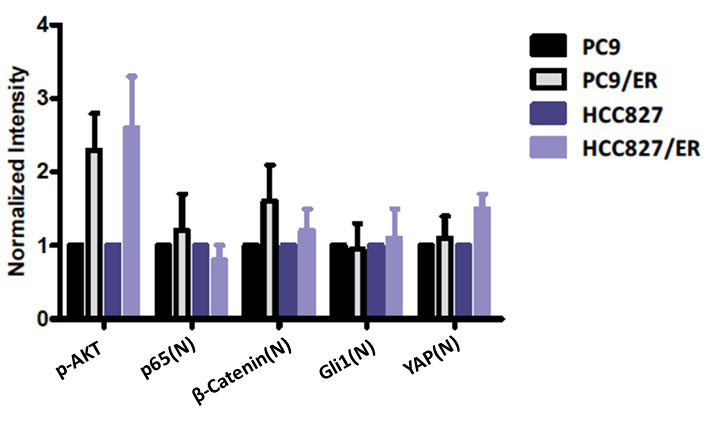

Supplement: Supplementary file 5 — Supplementary figure 4 [file 41419_2017_120_MOESM5_ESM.jpg]

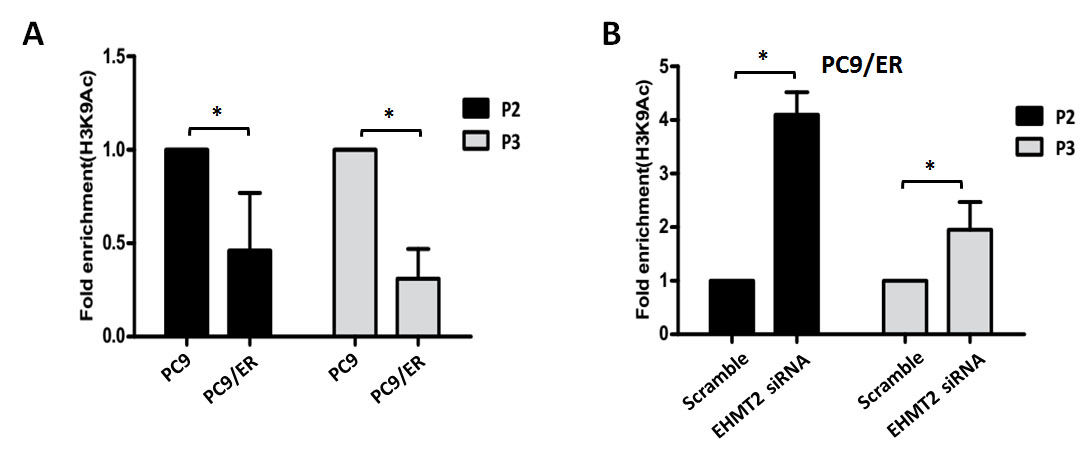

Supplement: Supplementary file 6 — Supplementary figure 5 [file 41419_2017_120_MOESM6_ESM.jpg]
