# Supplementary material for: Targeting EHMT2 reverses EGFR-TKI resistance in NSCLC by epigenetically regulating the PTEN/AKT signaling pathway
Source: Cell Death Dis. 2018 Jan 26;9(2):129. doi: 10.1038/s41419-017-0120-6 (PMC5833639; doi:10.1038/s41419-017-0120-6)
Supplement: Supplementary file 7 — Supplementary Figure Legends [file 41419_2017_120_MOESM7_ESM.docx]

**Supplementary Figure Legends**

**Supplementary Figure 1 Effects of epigenetic enzyme inhibitors on cell growth and apoptosis in EGFR-TKI-resistant NSCLC cells.**

(A) The growth of PC9 and HCC827 cells treated with UNC0638 at different concentrations (5 and 10 μM). Cell lines treated with DMSO were used as controls. (B) Quantification of cleaved PARP (Clv-PARP) expression in both PC9/ER and HCC827/ER cells using integral optical density analysis.

**Supplementary Figure 2 Apoptosis assessment after treated with various inhibitors in EGFR-TKI-resistant NSCLC cells.**

Cell apoptosis was assessed using Annexin V/PI double staining in resistant cells which were treated with UNC0638, PXD101, and JQ-1 at designed concentration (10 μM) for 48 h. Cell lines treated with DMSO were used as controls.

**Supplementary Figure 3 Effects of inhibiting EHMT2 or EHMT1 on the sensitivity of EGFR-TKI-resistant cells to TKIs.**

(A) Cell viability in EHMT2-knockdown PC9/ER cells treated with Erlotinib at different concentrations for 48 h. (B) Cell viability in EHMT1-inhibited PC9/ER cells treated with Erlotinib at different concentrations for 48 h. (C) Apoptosis assessment after treated with 1μM Erlotinib in PC9/ER cells with EHMT2 inhibition by siRNA (20 nM) or inhibitor UNC0638 (100 nM). Scramble siRNA-transfected or DMSO treated cells were used as controls.

**Supplementary Figure 4 Quantification of p-AKT, nuclear p65, nuclear β-Catenin, nuclear Gli1, and nuclear YAP expression levels in PC9/ER, HCC827/ER and parental cell lines using integral optical density analysis.** The proteins expression in parental cells were considered as 100%.

**Supplementary Figure 5 Enrichment of H3K9Ac in PC9 and PC9/ER cells.**

(A) ChIP assays were performed to measure the ability of H3K9Ac to bind to different promoter regions of the *PTEN* gene (P2 and P3) in PC9 and PC9/ER cells. (B) H3K9Ac enrichment was accessed in different *PTEN* gene promoter regions (P2 and P3) in PC9/ER cells with EHMT2 inhibition. * *P*<0.05, compared to corresponding control cells.
